# Bacterial communities varied in different *Coccinella transversoguttata* populations located in Tibetan plateau

**DOI:** 10.1038/s41598-024-65446-x

**Published:** 2024-06-26

**Authors:** Huanhuan Zhang, Kun Yang

**Affiliations:** 1 Tibet Academy of Agricultural and Animal Husbandry Sciences, Lhasa, 850032 Tibet People’s Republic of China; 2 Institute of Vegetable, Tibet Academy of Agricultural and Animal Husbandry Sciences, Lhasa, 850032 Tibet People’s Republic of China; 3 Shandong Province Centre for Bioinvasions and Eco-Security, Qingdao, 266109 People’s Republic of China; 4 Shandong Engineering Research Center for Environment-Friendly Agricultural Pest Managcment, College of Plant Health and Medicine, Qingdao Agricultural University, Qingdao, 266109 People’s Republic of China

**Keywords:** Bacterial community, Tibetan plateau, *Coccinella transversoguttata*, Altitude, Symbiont, Microbiology, Microbial communities, Entomology

## Abstract

*Coccinella transversoguttata* is an important predatory beetle in Asia and America. Currently, few studies have investigated *C. transversoguttata* in China especially in the Tibetan plateau. In this study, full-length 16 s rRNA sequencing and qPCR experiment were performed on eight *C. transversoguttata* populations collected from Tibet to analyze their bacterial communities and bacteria abundance. In summary, our results revealed the microbial compositions, diversities and bacterial titers in the bacterial communities in *C. transversoguttata* populations in the Tibetan plateau. In future, there is a need to explore the differences in microbiota among various *C. transversoguttata* populations collected from different locations. These results add to our understanding of the complex bacterial communities of *C. transversoguttata* and their utilization as potential biocontrol factors.

## Introduction

Globally, more than 6 000 ladybeetle species (Coccinellidae: Coleoptera) have been reported^1^. Although *Coccinella transversoguttata* is a ladybeetle native to North America, it has spread to many other areas including Central America, Mexico, Europe as well as the Tibetan plateau in China^2–7^. *C. transversoguttata* has been found to be an important predatory ladybug that feeds on many agricultural pest including aphids, scale insects and other small arthropods^8–10^. In many locations including America, *C. transversoguttata* is considered a notable predator that can be exploited in the biological control of many pests mainly aphids^11,12^.

In Tibet area, which has the highest plateau in the world, many agricultural pest species have been reported including aphids, whiteflies, phylloxeras, scale insects, spider mites and many other pests. They cause enormous losses in terms of crop yield in the Tibet area^13–17^. *Metopolophium dirhodum* and *Rhopalosiphum padi* were reported to be the dominant aphid species in Tibet area farmland of highland barley which are difficultly to control^15^. Besides *C. transversoguttata,* several other predatory ladybeetles exist in Tibet such as *Hippodamia variegate*, *Harmonia axyridis*, *Coccinella septempuntata,* which may be used as biotic factors to control agricultural pests in Tibet plateau, especially aphids^5^. This calls for development of strategies to improve the control efficiency of predatory ladybeetles on pests.

Bacterial communities have important regulatory roles in the growth, reproduction, digestion, thermotolerance, resistance to adverse factors and other important processes in the host insects^18–22^ as well as other arthropods including spider mites^23–25^. In *Drosophila melanogaster*, more than 10 different lysozymes have been detected in the midgut; which also harbors a transporter with high affinity for d-amino acids^26^. In crickets, bacteria in the anterior hindgut degrade various classes of soluble polysaccharides^27^. Another study showed that the bacterial community in bumblebees protect the host against highly virulent natural parasite (*Crithidia bombi*)^28^.

The microbiome of Coleoptera encompasses a complex assemblage of mainly bacteria and other microorganisms inhabiting various niches within and on the beetle's body, including the gut, integument, and reproductive organs, which play diverse and often essential roles in beetle biology, influencing nutrient acquisition, digestion, immunity, reproduction, and defense against pathogens and predators^18,29,30^, Understanding the microbiome of Coleoptera is essential for unraveling the intricate interactions between beetles and their microbial partners, shedding light on fundamental ecological and evolutionary processes.

Currently, few studies have characterized *C. transversoguttata* in aphids in Tibet areas. In this study, we collected *C. transversoguttata* from 8 locations in the Tibetan plateau and found that feeding habits affected the structure and diversity of bacterial communities in beetles. However, we did not identify the factor that influence the bacterial structures. These results add to our understanding of the complex bacterial communities of *C. transversoguttata* and their utilization as potential biocontrol factors.

## Materials and methods

### *Coccinella* transversoguttata population collected in Tibetan area

Eight *C. transversoguttata* populations were collected from Tibetan plateau, each population contains 3 replicates, and every replicate includes combined 5 *C. transversoguttata* adults, in the range of latitude N 29.07°–N 30.01° and longitude E 88.28°–E 96.69°, and all the samples were collected at an altitude of 2 942 m above sea level. The host plants of *C. transversoguttata* were mainly apple, highland barley and alfalfa, and many aphids in these plants can be preyed by *C. transversoguttata* such as *Aphis gossypii*, *Macrosiphum avenae, Schizaphis graminum, Acyrthosiphon dirhodum* (Table 1). Once *C. transversoguttata* were collected, they were put in abosolute alcohol until sequencing. Total *C. transversoguttata* adults were used for 16 s rRNA sequencing without dissection, as microbiota could exist within any organ of insects, including gut, gonad, salivary glands or cells. Before sequencing, all *C. transversoguttata* adults were washed by 75% alcohol to wipe off the microbe in the surface of *C. transversoguttata*.

**Table 1:** Information about *Coccinella transversoguttata* populations collected in Tibetan plateau.

Population name	Collection location	Latitude	Longitude	Altitude (m)	Host plant	Potential prey
BS	Basu country, Chamdo city, Tibet	N 30.01°	E 96.69°	3764.20	Apple	*Aphis gossypii*
GC	Gyaca County, Shannan city, Tibet	N 29.08°	E 92.73°	3177.60	Highland barley	Macrosiphum avenae, Schizaphis graminum, Acyrthosiphon dirhodum
LS	Lasa city, Tibet	N 29.64°	E 91.04°	3641.41	Highland barley	
MLgmnc	Karma farm, Mainling country, Linzhi city, Tibet	N 29.42°	E 94.44°	2942.00	Apple	*Aphis gossypii*
MLrc	Ri village, Linzhi city, Tibet	N 29.07°	E 93.38°	3041.70	Apple	*Aphis gossypii*
MR	Mirui country, Linzhi city, Tibet	N 29.48°	E 94.56°	2942.40	Apple	*Aphis gossypii*
SH	Shigatse city, Tibet	N 29.28°	E 88.28°	3836.40	Apple	*Aphis gossypii*
ZN	Zhanang country, Shannan city, Tibet	N 29.26°	E 91.27°	3558.40	Alfalfa	Spotted alfalfa aphid

### Full-length 16 s rRNA sequencing of 8 *C. transversoguttata* populations

To identify differences in microbial communities among different *C. transversoguttata* populations, full–length 16 s rRNA gene sequencing was performed. Notably, *C. transversoguttata* DNA amplification, sequencing, library construction, and 16 s rDNA data analysis were carried out using the Biomarker Technologies Corporation, Beijing, China. DNA extract of *C. transversoguttata* were performed by TaKaRa MiniBEST Universal Genomic DNA Extraction Kit Ver.5.0 with instructions by TaKaRa Co., Ltd. (Dalian, China). Briefly, the V3–V4 region of the whitefly’s bacterial 16 s rRNA gene was first amplified with a pair of primers: forward primer 27f. (5′-ACTCCTACGGGAGGCAGCA-3′) and reverse primer 1492r (5′-GGACTACHVGGGTWTCTAAT-3′). In this test, a combination of barcode sequences and adapter sequences was implemented. The PCR amplification experiment was carried out as previously described^21^, and Quant–iT™ dsDNA HS Reagent was used to quantify the PCR products. Next, all products were pooled and sequenced on the sequencing platform of PacBio SMRT RS II DNA (Pacific Biosciences, Menlo Park, CA, USA) to construct a sequence amplified library. Off–target sequences and low–quality sequences were filtered using the PacBio circular consensus sequencing technology^31^. The raw reads generated from sequencing were filtered and demultiplexed using the SMRT Link software (version 8.0) (https://www.pacb.com/support/software-downloads/) with the minPasses ≥ 5 and minPredictedAccuracy ≥ 0.9, in order to obtain the circular consensus sequencing (CCS) reads. Subsequently, the lima (version 1.7.0) was employed to assign the CCS sequences to the corresponding samples based on their barcodes. CCS reads containing no primers and those reads beyond the length range were discarded through the recognition of forward and reverse primers and quality filtering using the Cutadapt quality control process (version 2.7). The UCHIME algorithm (v8.1) was used in detecting and removing chimera sequences to obtain the clean reads. Sequences with similarity ≥ 97% were clustered into the same operational taxonomic unit (OTU) by USEARCH (v10.0), and the OTUs with rebundace < 0.005% were filtered. Unassigned OTU refers to taxa that cannot be matched to the database, are not annotated, or are unclear in terms of species identification. Others OTU refers to species with lower abundances.

The phylogenetic tree; diversity indexes, including alpha diversity (Ace, Chao1, Shannon, and Simpson) and beta diversity indexes (PCoA analysis and heatmaps based on Bray–Curtis similarity analysis); and function of bacterial communities (analyzed by PICRUSt, Phylogenetic Investigation of Communities by Reconstruction of Unobserved States) were all analyzed using the Usearch, mothur v1.30.0 (to obtain the OTU and taxonomy matrices), and QIIME v2.0 software (https://qiime2.org/) on the BMK Cloud (www.biocloud.net) (to analyze the beta diversity indexes). The Shapiro–Wilk test (SPSS 21.0) was conducted to assess whether the alpha diversity index data followed a normal distribution. Given that the data exhibited a normal distribution, Student's t-test (SPSS 21.0) was employed to analyze the significant differences in symbiont density between the two treatment groups.

### qPCR experiment of *bacteria* in 8 C. transversoguttata populations

The qPCR primers of bacteria in *C. transversoguttata* were designed using Primer Premier 6.0 based on the 16 s rRNA gene sequence. GAPDH gene served as the reference gene in *C. transversoguttata* (Table S1). qTOWER 2.0/2.2 Real Time PCR Systems (Jena Bioscience GmbH, Thüringen, Germany) with SYBR Premix Ex Taq (Takara Bio Inc., Dalian, China) were utilized to run the qPCR reactions. Regarding the qPCR results of bacteria abundance in *C. transversoguttata* (Fig. 6), if the data exhibited normal distribution, one–way ANOVA with post-hoc Tukey HSD analysis was performed. If the bacteria abundance and diversity data did not conform to normal distribution, they were analyzed using the Kruskal–Wallis test and Dunn’s test with Bonferroni correction for multiple comparisons. All statistical analyses were performed using SPSS 21.0. All figures were drafted using GraphPad Prism 9.0.0.

## Results

### Abundance of different *bacteria* in various C. transversoguttata populations

**Figure 1:**
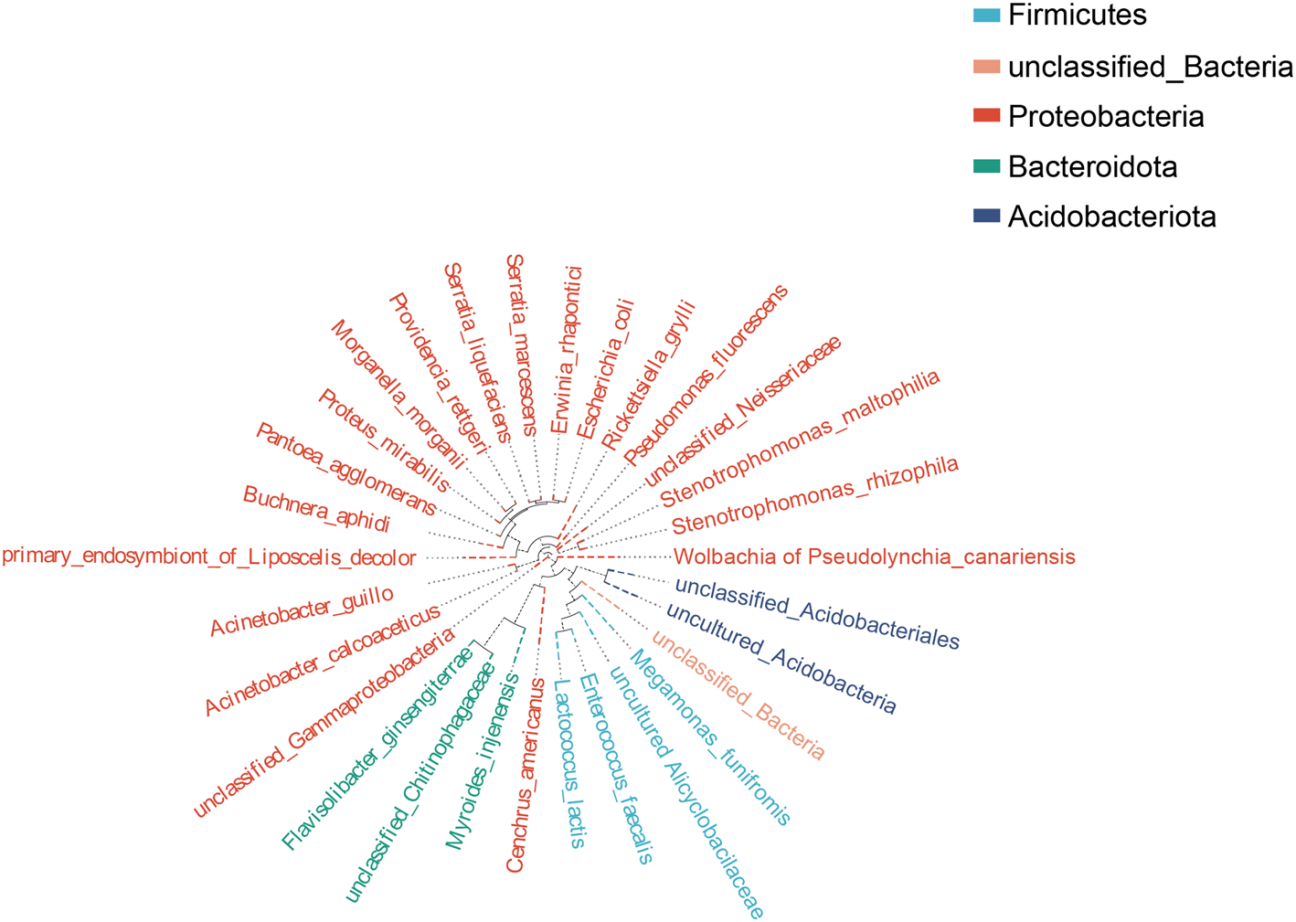
Phylogenetic tree of all detected bacteria in different *Coccinella transversoguttata* populations in Tibetan plateau by full-length 16S rRNA genes based on Probabilistic Methods of Phylogenetic Inference constructed by FastTree 2.0.0 software (http://www.microbesonline.org/fasttree). Bacteria related to different phylum are in different colors.

**Figure 2:**
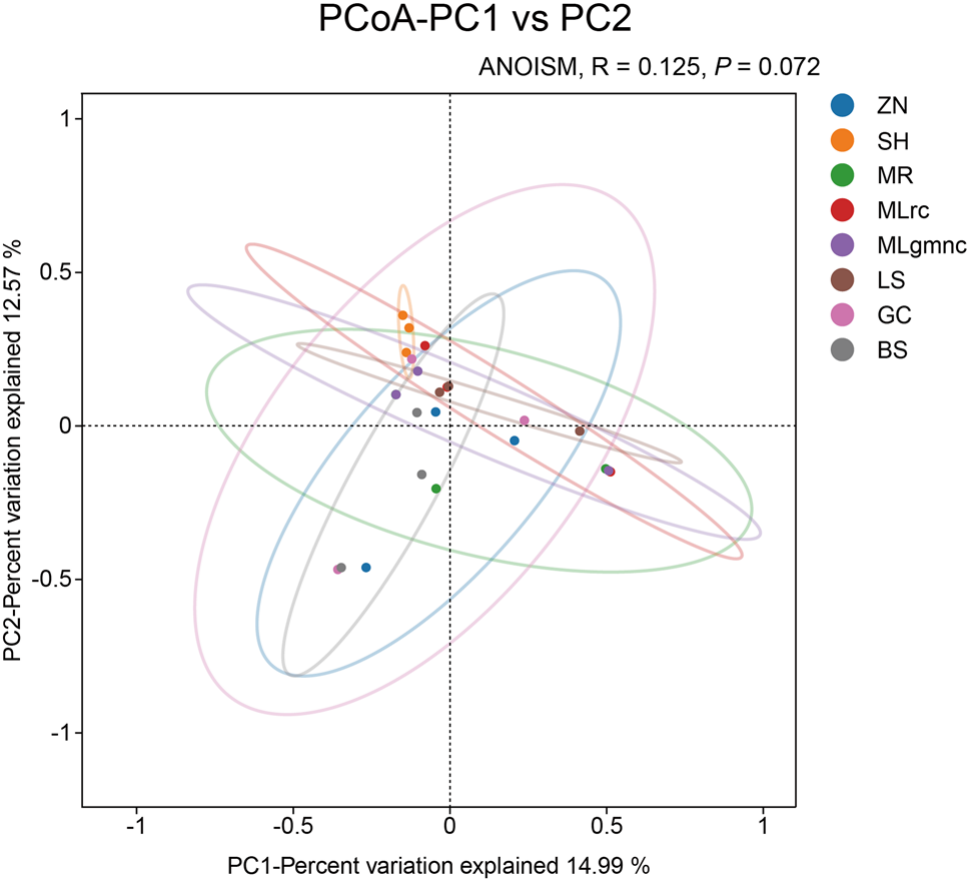
Beta diversity analysis of bacterial communities in different *Coccinella transversoguttata* populations in Tibetan plateau, the results of PCA analysis was shown.

**Figure 3:**
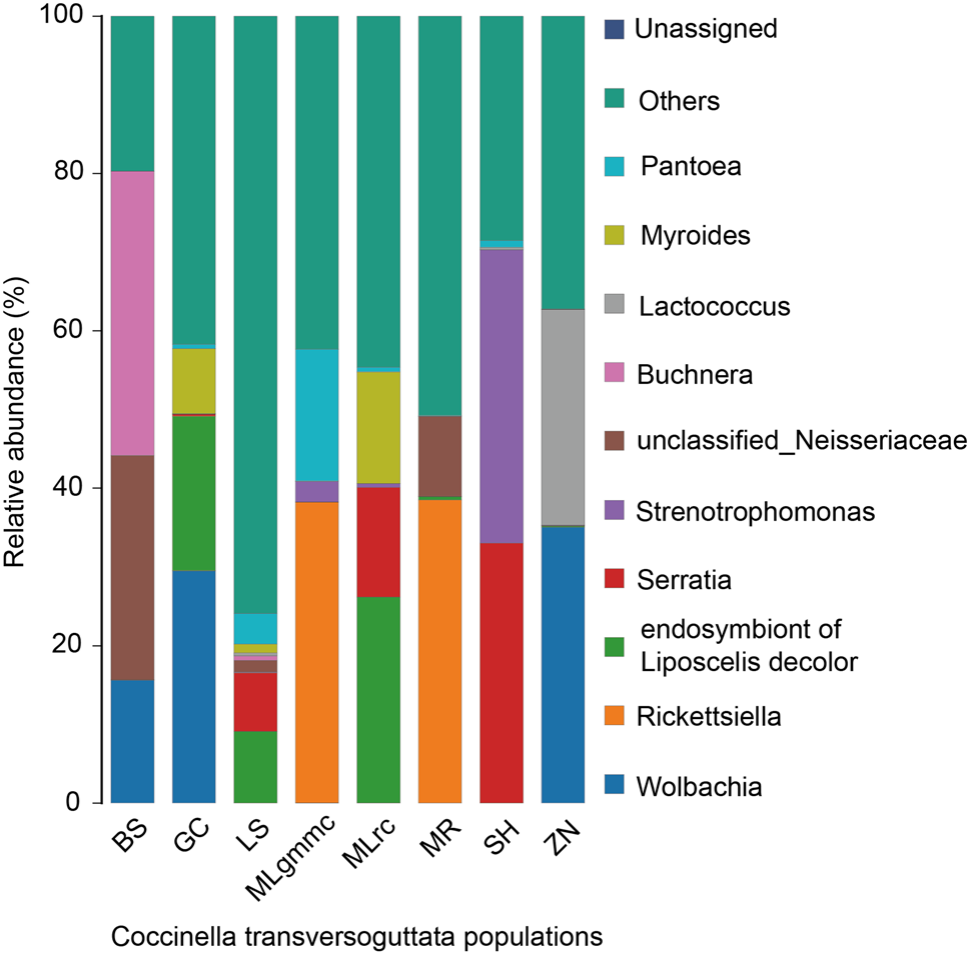
Relative abundance of top 10 bacterial genera in Tibetan plateau *Coccinella transversoguttata* by full-length 16 s rRNA gene sequencing.

Sequencing data of the 16S rRNA genes of eight *C. transversoguttata* populations were shown in Table 2. In total, 32 bacteria species were detected in all *C. transversoguttata* populations, and most of them belonged to the filum Proteobacteria (Fig. 1). PCoA analysis revealed no significant differences among all the bacterial communities in all *C. transversoguttata* populations (Fig. 2). In terms of bacterial genus abundance, *Wolbachia* was detected in three populations (BS, GC and ZN), *Serratia* was found in three populations (LS, MLrc and SH) and Rickettsia was found in MLgmmc and MR populations (Fig. 3).

**Table 2:** Full-length 16 s rRNA sequencing results of all *Coccinella transversoguttata* samples collected in Tibetan plateau.

Sample ID	Raw CCS	Clean CCS	Effective CCS	AvgLen (bp)	Effective (%)	Species
BS1	12,652	12,634	12,632	1472	99.84	83
BS2	13,100	13,068	12,999	1457	99.23	735
BS3	10,191	10,180	10,087	1442	98.98	129
GC1	10,564	10,522	10,518	1429	99.56	62
GC2	11,178	11,168	11,025	1459	98.63	179
GC3	14,351	14,284	13,972	1449	97.36	695
LS1	12,096	12,079	12,044	1458	99.57	652
LS2	12,812	12,797	12,696	1456	99.09	667
LS3	14,728	14,711	14,359	1454	97.49	803
MLgmnc1	10,346	10,329	10,265	1460	99.22	417
MLgmnc2	14,202	14,183	13,835	1448	97.42	796
MLgmnc3	14,109	14,109	14,109	1484	100	26
MLrc1	10,894	10,887	10,872	1451	99.8	116
MLrc2	12,455	12,454	12,165	1458	97.67	145
MLrc3	12,171	12,155	11,814	1448	97.07	778
MR1	12,069	12,069	12,069	1485	100	6
MR2	8347	8340	8229	1453	98.59	441
MR3	14,514	14,502	14,138	1448	97.41	741
SH1	11,722	11,706	11,704	1466	99.85	111
SH2	11,078	11,065	10,897	1467	98.37	160
SH3	12,638	12,635	12,213	1467	96.64	392
ZN1	9276	9209	8923	1461	96.19	364
ZN2	13,778	13,778	13,706	1428	99.48	209
ZN3	12,928	12,925	12,832	1464	99.26	524

CCS: circular consensus sequences generated by PacBio platform; Raw CCS: Counts of identified CCS reads in the sample; Clean CCS: Counts of clean CCS reads (post primer removal and length filtration); Effective–CCS: Counts of effective CCS reads after chimeric reads removal; Avglen (bp): Average length reads in the sample; Effective (%): Percentage of effective CCS reads in raw reads.

### Bacterial diversity and functional analysis in *C. transversoguttata* populations

**Figure 4:**
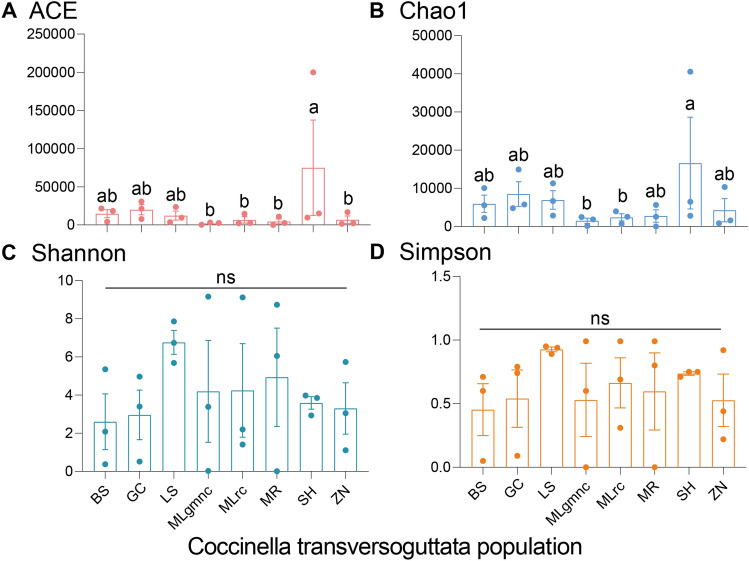
Alpha diversity index of bacterial communities in different *Coccinella transversoguttata* populations in Tibetan plateau, the four diversity indices including ACE (**A**), Chao1 (**B**), Shannon (**C**) and Simpson (**D**) were shown, respectively.

**Figure 5:**
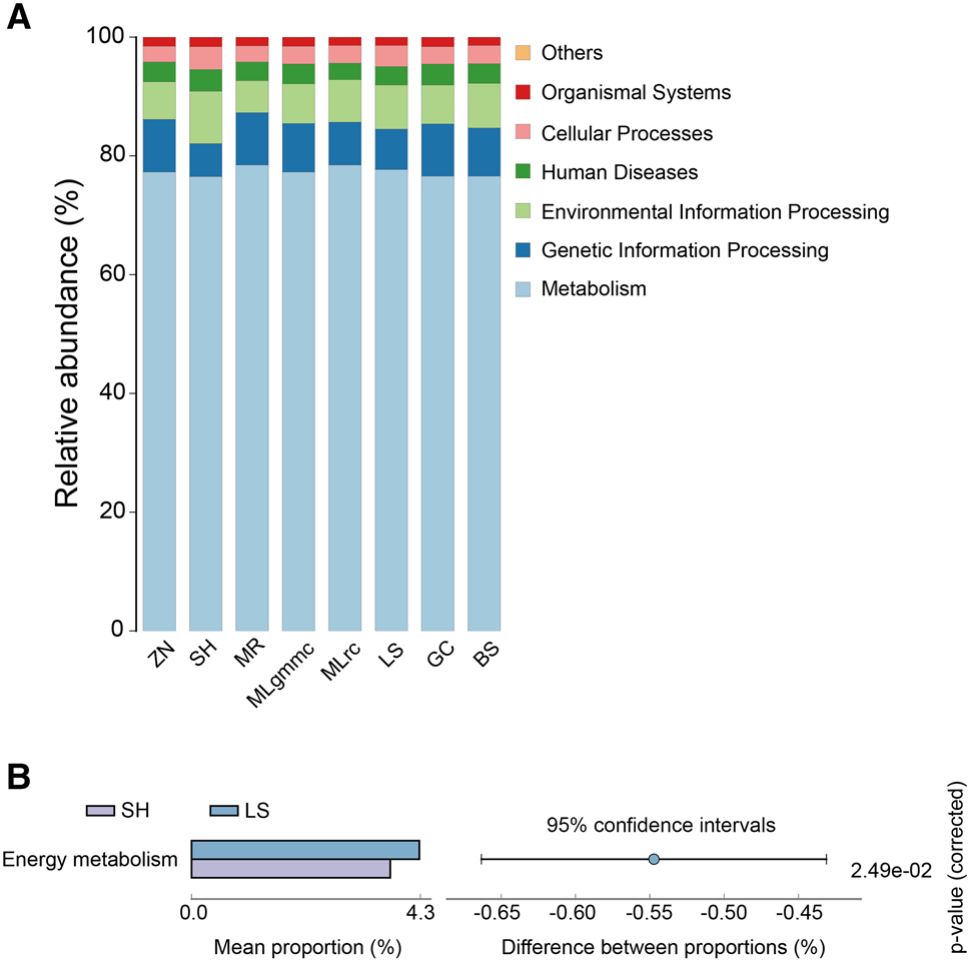
Functional analysis of bacteria in *Coccinella transversoguttata*. (**A**) Functional analysis of bacterial communities in different *Coccinella transversoguttata* populations in Tibetan plateau based on PICRUSt (Phylogenetic Investigation of Communities by Reconstruction of Unobserved States) at BMK Cloud (www.biocloud.net); (**B**) significant differences of functional analysis of bacterial communities between SH and LS populations.

The alpha diversity indices of bacterial communities in all *C. transversoguttata* populations were compared using One–Way ANOVA in SPSS 21.0. The results revealed significantly higher ACE and Chao1 indices in the SH population compared to the MLgmmc, MLrc, MR, and ZN populations, while the Shannon and Simpson indices were similar across all populations (Fig. 4). Functional analysis revealed that genes related to metabolism were the most enriched in all bacterial communities of *C. transversoguttata* populations (Fig. 5A). In comparison, the LS population had significantly higher levels of genes involved in energy metabolism than the SH population (Fig. 5B).

### *Bacteria* abundance varied among different C. transversoguttata populations

**Figure 6:**
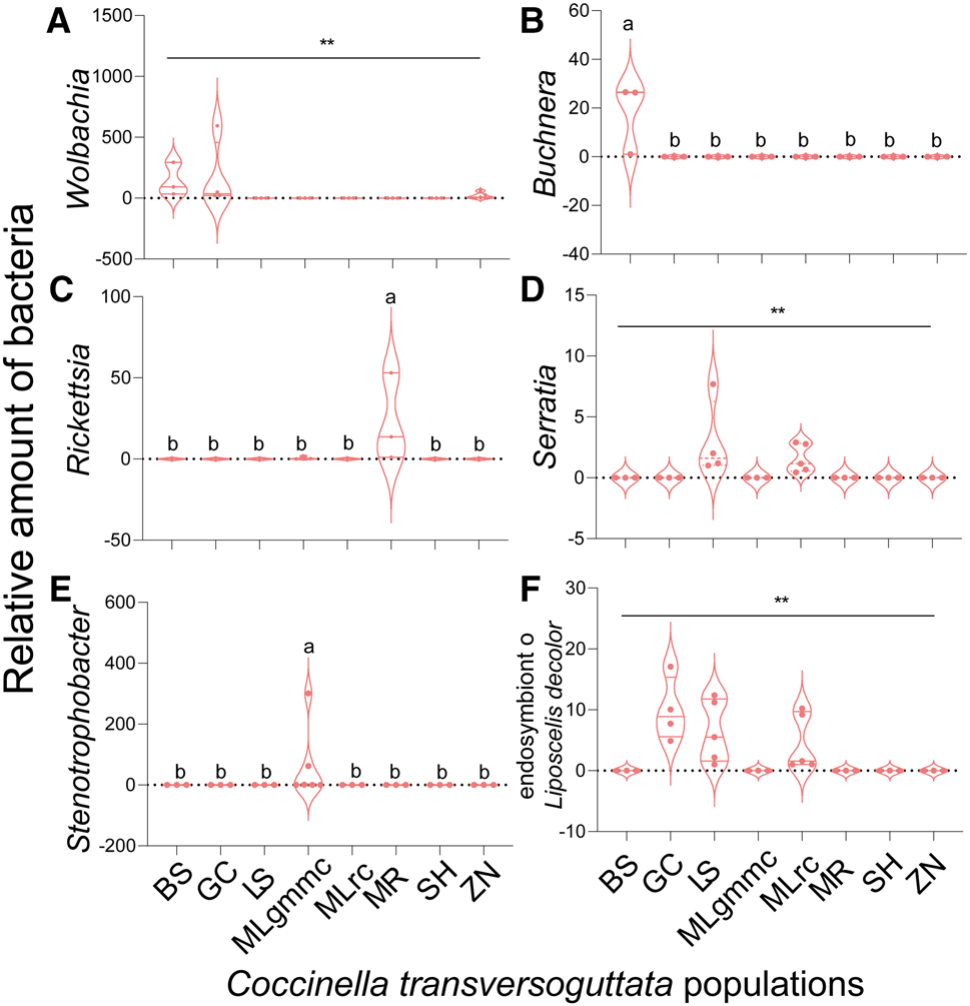
Relative amount of symbiont density in different *Coccinella transversoguttata* populations in Tibetan plateau, a total of 6 bacteria including *Wolbachia* (**A**), *Buchnera* (**B**), *Rickettsia* (**C**), *Serratia* (**D**), *Stenotrophobacter* (**E**) and *endosymbiont of Liposcelis decolor* (**F**) were shown.

Analysis of the qPCR results revealed that the *Wolbachia* abundance varied significantly different among the *C. transversoguttata* populations (*P* < 0.01, Kruskal–Wallis tests), while the post–hoc of Dunn’s tests with Bonferroni correction indicated found no significant difference between any 2 populations (Fig. 6A). The abundance of *Buchnera* in BS population (17.97 ± 8.48, Mean ± SEM) was significantly higher than that in other populations (*P* < 0.01, Kruskal–Wallis tests) (Fig. 6B). The abundance of *Rickettsia* in MR population (22.61 ± 15.69, Mean ± SEM) was significantly higher compared with that in other populations (*P* < 0.05, Kruskal–Wallis tests) (Fig. 6C). The *Serratia* abundance varied significantly different among the *C. transversoguttata* populations (*P* < 0.01, Kruskal–Wallis tests), but post–hoc of Dunn’s tests with Bonferroni correction indicated that there was no significant difference among the paired comparisons (Fig. 6D). The abundance of *Stenotrophobacter* in MLgmmc population (60.87 ± 49.04, Mean ± SEM) was significantly higher relative to that of other populations (*P* < 0.05, Kruskal–Wallis tests) (Fig. 6E). Notably, the abundance of endosymbiont *of Liposcelis decolor* varied significantly among the *C. transversoguttata* populations (*P* < 0.01, Kruskal–Wallis tests), while the post–hoc of Dunn’s tests with Bonferroni correction found no significant difference in the paired comparisons (Fig. 6F).

## Discussion

In this study, we explored the influence of different locations on bacterial communities of *C. transversoguttat* in Tibetan plateau. Through full-length 16S rRNA gene sequencing and qPCR experiment, it is to be noticed that microbiome of 8 *C. transversoguttata* populations were complex and abundant. The OTU number and alpha–diversity index of different *C. transversoguttat* populations bacterial communities were diverse. The abundance of bacteria, including many symbionts, was significantly different among all *C. transversoguttat* populations. Factors contributing to this phenomenon in *C. transversoguttat* need to be further explored.

In many host insects, some bacteria are considered essential, and hence are widely spread among hosts. For example, Gammaproteobacteria are considered as essential primary symbiont in the pentatomid bug *Graphosoma lineatum* because they have important regulatory roles in the host biology^32^. *Wolbachia* exhibited mitochondrion–like function in some nematodes, generating ATP for their hosts^33^, in other Coleoptera species dominate bacteria were also detected, such as *Spiroplams* would dominate the microbiome of khapra beetle, *Trogoderma granarium*^34^, while *Wolbachia* and *Rickettsia* were dominated in cereal leaf beetle, *Oulema melanopus*^35^. However, in this study, although numerous bacteria were detected in many *C. transversoguttata* populations (such as *Serratia*, *Wolbachia* and *Rickettsia*), no dominant or essential bacteria were observed. In contrast to nematodes, *Wolbachia* or *Spiroplasma* were not essential for *T. truncatus*. These results are similar to those observed in *Drosophila*: *Drosophila* species harbor diverse microbiota^36^.

To date, researchers have explored factors influencing bacterial community of insects. Host diet is regarded as an important factor affecting the structure of insect gut bacterial communities^37^. Rearing environment has also been shown to structure microbial communities in *Tenebrio molitor*^38^. Host-endosymbiont interactions are regulated by environmental factors, including climatic and other geographical factors ^39^. Geographic distance may also be an important factor affecting the bacterial community structure. It has been shown that the bacterial community structure was similar within *Aphis gossypii* obtained from the same province, but it was distinct among those from different provinces, indicating a strong effect of geographic distance on aphid bacterial communities^40^. In the study, despite the varied diets observed among different populations of *C. transversoguttata* (Table 1), the available data alone are insufficient to ascertain the significance of dietary effects on the microbiome of *C. transversoguttata*.

In summary, our results revealed the microbial compositions, diversities and bacterial titers of bacterial communities in different *C. transversoguttata* populations in Tibetan plateau. Further studies are advocated to explore differences in microbial communities including symbionts among different *C. transversoguttata* populations collected from different locations. These results expand our understanding of the complex bacterial communities in *C. transversoguttata* and provide ideals to accelerate the utilization of *C. transversoguttata* as a potential biocontrol factor.

### Supplementary Information

Supplementary Information.

## Data Availability

Sequence data that support the findings of this study have been deposited in the NCBI with the primary accession code SUB14356154. All data generated or analyzed during this study are included in this article.
