# Supplementary material for: Bacterial communities varied in different Coccinella transversoguttata populations located in Tibetan plateau
Source: Sci Rep. 2024 Jun 26;14:14708. doi: 10.1038/s41598-024-65446-x (PMC11208169; doi:10.1038/s41598-024-65446-x)
Supplement: Supplementary file 1 — Supplementary Information. [file 41598_2024_65446_MOESM1_ESM.docx]

**
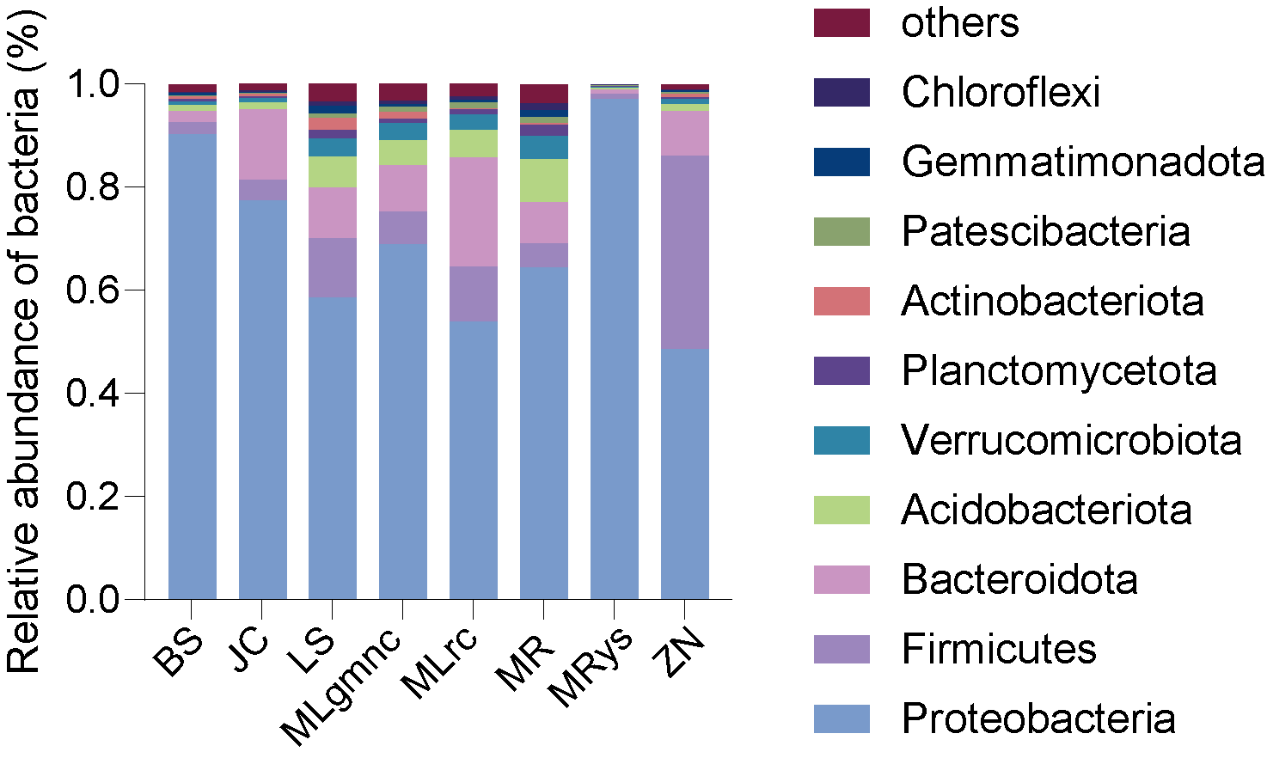
**

**Figure S1.** Relative abundance of top 10 bacterial phylum in Tibetan plateau *Coccinella transversoguttata* by full-length 16s rRNA gene sequencing.


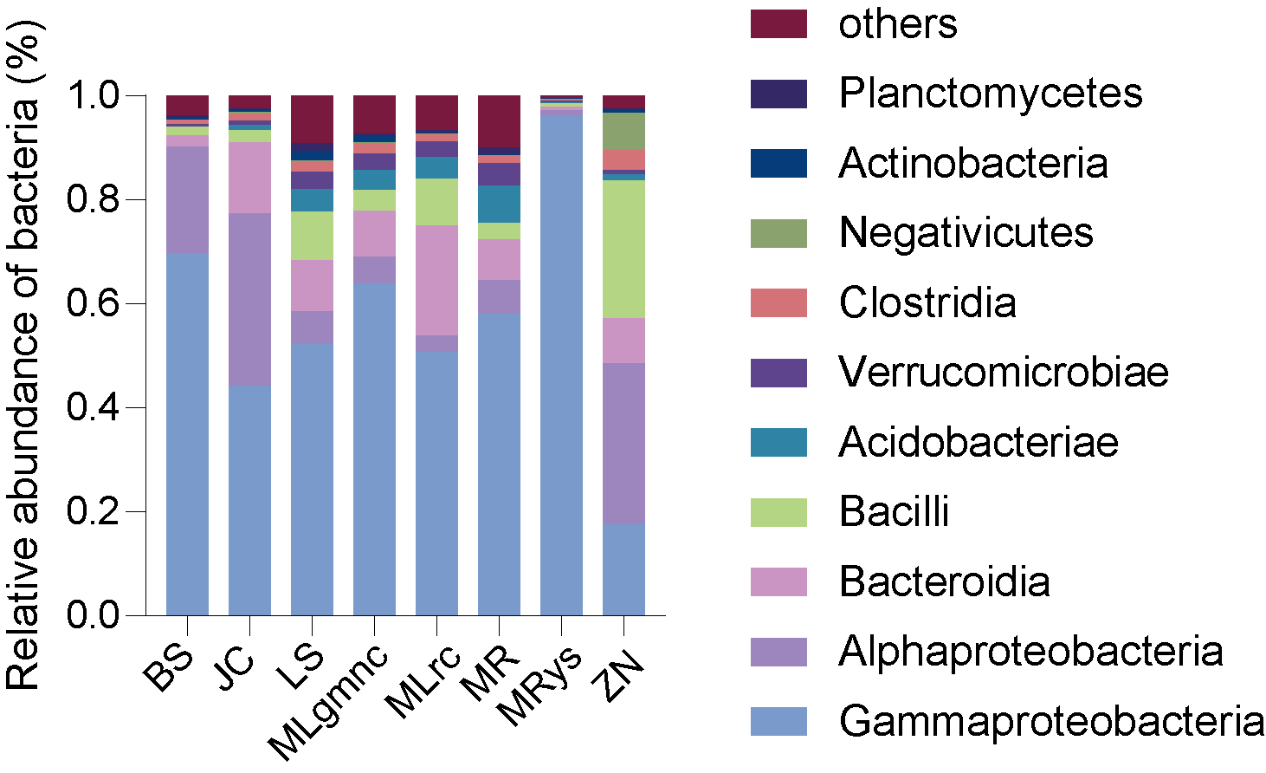


**Figure S2.** Relative abundance of top 10 bacterial classes in Tibetan plateau *Coccinella transversoguttata* by full-length 16s rRNA gene sequencing.

**
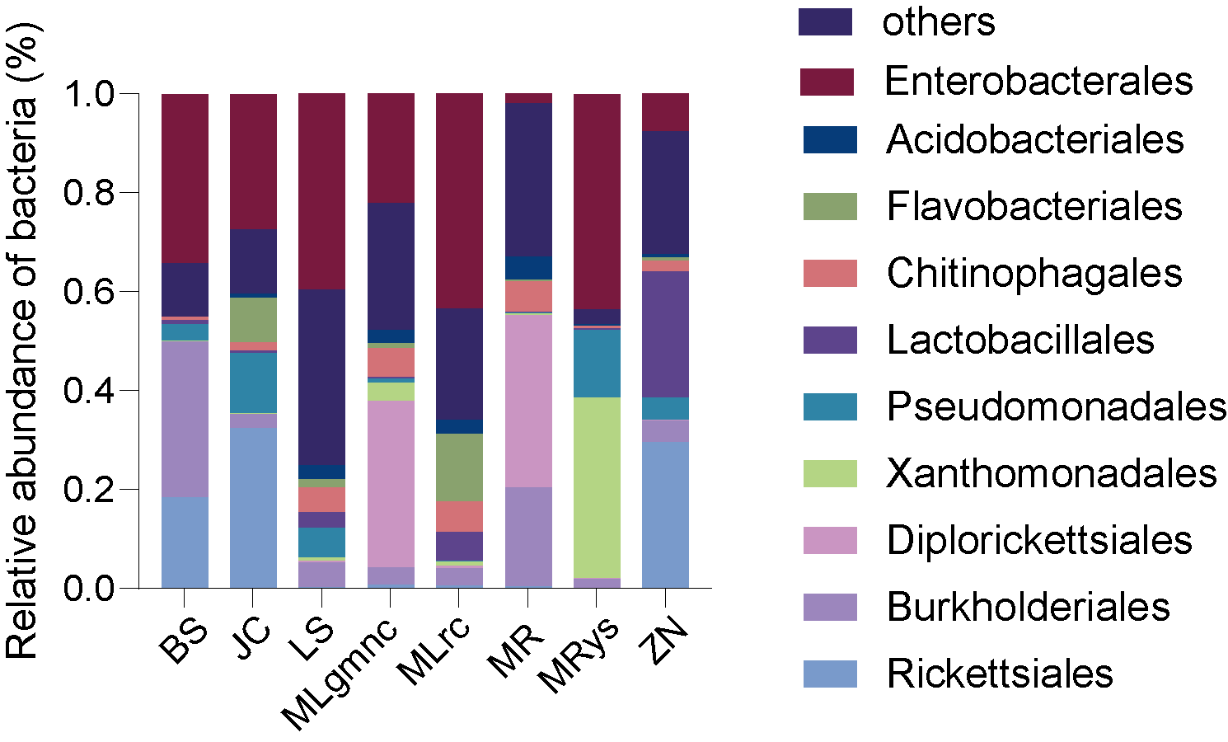
**

**Figure S3.** Relative abundance of top 10 bacterial orders in Tibetan plateau *Coccinella transversoguttata* by full-length 16s rRNA gene sequencing.


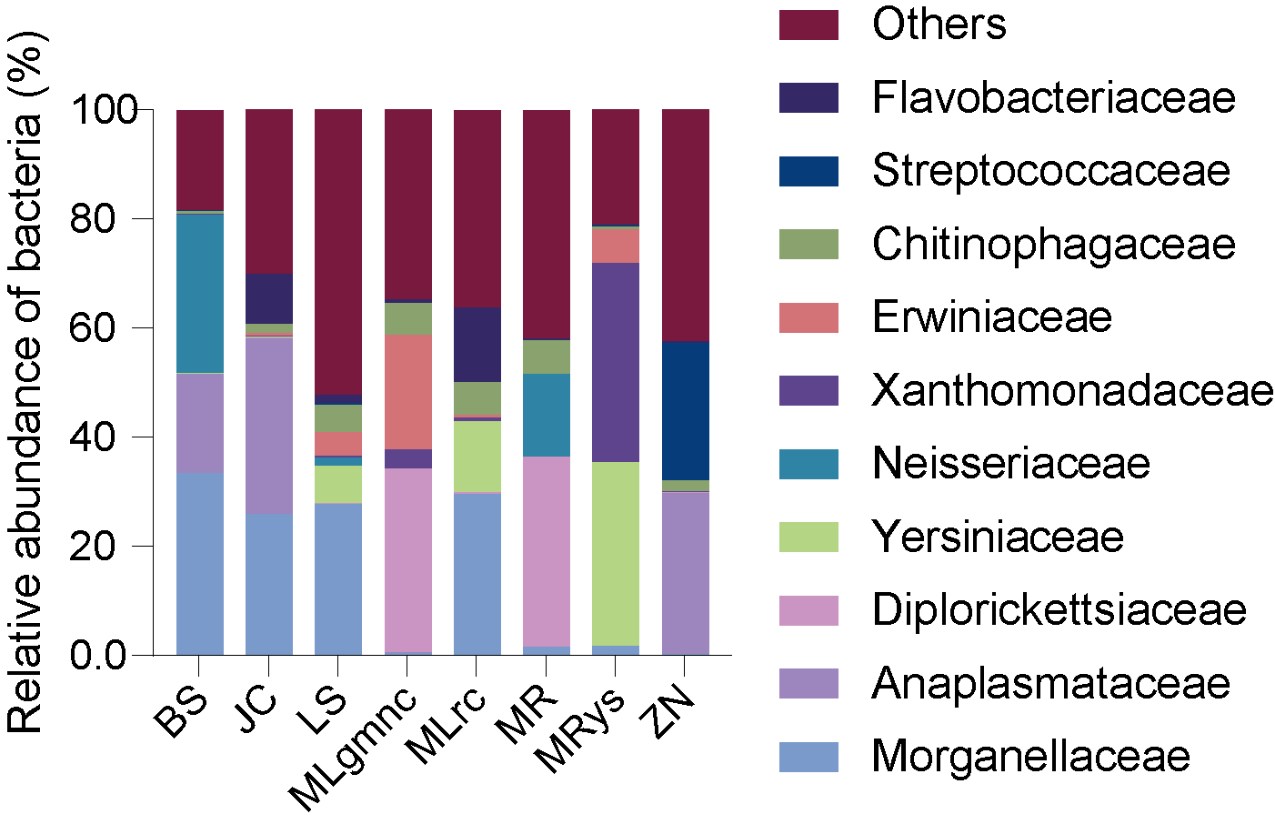


**Figure S4.** Relative abundance of top 10 bacterial families in Tibetan plateau *Coccinella transversoguttata* by full-length 16s rRNA gene sequencing.


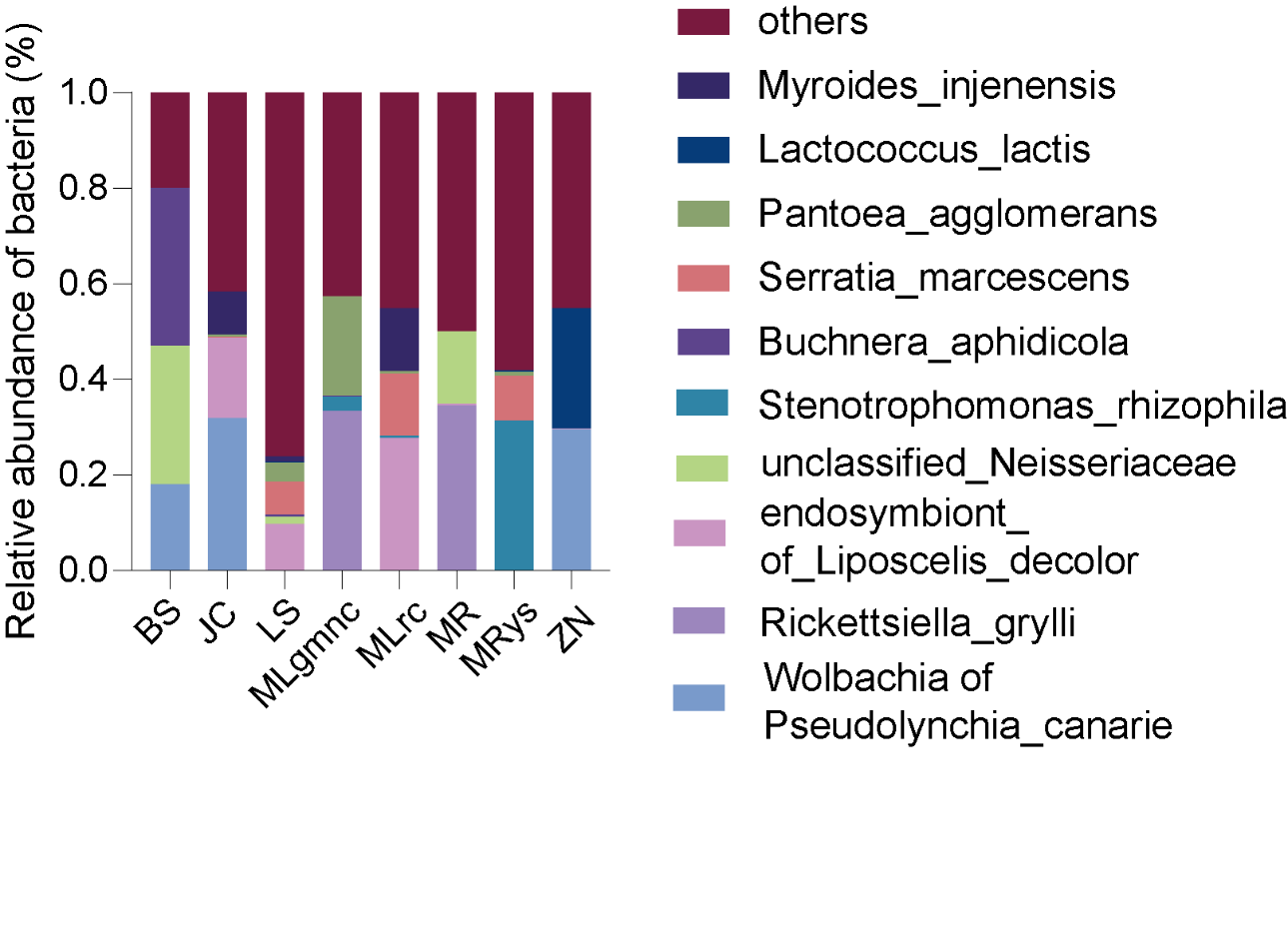


**Figure S5.** Relative abundance of top 10 bacterial species in Tibetan plateau *Coccinella transversoguttata* by full-length 16s rRNA gene sequencing.

**Table S1.** Primers used in qPCR experiment of bacteria in different *Coccinella transversoguttata* populations in Tibetan plateau.

| Primer name | Sequence (5’-3’) |
| --- | --- |
| Wolbachia-F | TCAGCCACACTGGAATTGAGATACG |
| Wolbachia-R | GCACGGAGTTAGCCAGGACTTC |
| Rickettsiella-F | ACGATGAGAACTGGCTGTGATATGT |
| Rickettsiella-R | CCAAGGATGTCAAGGGTAGGTAAGG |
| endosymbionts-F | GTGCTACAATGGTGCGTACAGAGG |
| endosymbionts-R | GCGATTACTAGCGATTCCGACTTCA |
| Buchnera-F | AAGTGACTTCCGAAGCTAACGCATT |
| Buchnera-R | CTGTGGATGTCAAGACCAGGTAAGG |
| Stenotrophomonas-F | AAGCGTGCGTAGGTGGTTGTT |
| Stenotrophomonas-R | TCGTGCCTCAGTGTCAGTGTTG |
| Serratia-F | AGACACGGTCCAGACTCCTACG |
| Serratia-R | CACCACCTTCCTCCTCGCTGAA |
| GAPDH-F | TCCCATGTATGTCTGTGGTGTCA |
| GAPDH-R | TGGTGGTGCAGGAAGCATTG |
